# Changes in Alcohol Consumption during the COVID-19 Pandemic Are Dependent on Initial Consumption Level: Findings from Eight European Countries

**DOI:** 10.3390/ijerph181910547

**Published:** 2021-10-08

**Authors:** Ingeborg Rossow, Miroslav Bartak, Kim Bloomfield, Fleur Braddick, Elin K. Bye, Carolin Kilian, Hugo López-Pelayo, Pia Mäkelä, Inger Synnøve Moan, Jacek Moskalewicz, Benjamin Petruzelka, Vladimír Rogalewicz, Jakob Manthey

**Affiliations:** 1 Department of Alcohol, Tobacco and Drugs, Norwegian Institute of Public Health, 0213 Oslo, Norway; ElinKristin.Bye@fhi.no (E.K.B.); IngerSynnove.Moan@fhi.no (I.S.M.); 2 Department of Addictology, First Faculty of Medicine and General University Hospital in Prague, Charles University, 12800 Praha, Czech Republic; miroslav.bartak@lf1.cuni.cz (M.B.); benjamin.petruzelka@lf1.cuni.cz (B.P.); vladimir.rogalewicz@lf1.cuni.cz (V.R.); 3 Centre for Alcohol and Drug Research, Aarhus University, 2400 Copenhagen, Denmark; kb.crf@psy.au.dk; 4 Clínic Foundation for Biomedical Research (FCRB), 08037 Barcelona, Spain; fleur.braddick@gmail.com; 5 Institute of Clinical Psychology and Psychotherapy, Technische Universität Dresden, 01187 Dresden, Germany; carolin.kilian@tu-dresden.de (C.K.); jakob.manthey@tu-dresden.de (J.M.); 6 Clinical Addictions Research Group (GRAC-GRE), Psychiatry Department, Neurosciences Institute, Hospital Clínic, Institut d’Investigacions Biomèdiques August Pi i Sunyer, University of Barcelona, 08037 Barcelona, Spain; HLOPEZ@clinic.cat; 7 Finnish Institute for Health and Welfare, 00271 Helsinki, Finland; pia.makela@thl.fi; 8 Institute of Psychiatry and Neurology, 02-957 Warsaw, Poland; moskalew@ipin.edu.pl; 9 Centre for Interdisciplinary Addiction Research, University Medical Center Hamburg-Eppendorf, 20246 Hamburg, Germany; 10 Department of Psychiatry, Medical Faculty, University of Leipzig, 04103 Leipzig, Germany

**Keywords:** alcohol consumption, COVID-19, restrictions, polarization, heavy drinking, comparison, Europe

## Abstract

Evidence suggests that changes in alcohol consumption during the first months of the COVID-19 pandemic were unevenly distributed over consumer groups. We investigated possible inter-country differences in how changes in alcohol consumption are contingent on initial consumption (before or at the start of the pandemic), and how changes in consumption translate into possible changes in the prevalence of heavy drinking. We used data from the European Survey on Alcohol use and COVID-19 (ESAC) conducted in Czechia, Denmark, Finland, Germany, Norway, Poland, Spain, and the UK (N = 31921). Past-year alcohol consumption and changes in consumption were measured by AUDIT-C. Drinking habits were compared according to percentiles of pre-pandemic consumption levels, below versus above the 90th percentile. Across countries, drinkers in the highest 10% for pre-pandemic consumption increased their drinking during the pandemic, whereas absolute changes among those initially drinking below this level were modest. The percentage of people reporting >28 alcohol units/week increased significantly in seven of eight countries. During the first months of the COVID-19 pandemic, alcohol consumption in the upper decile of the drinkers increased as did the prevalence of heavy drinkers, in contrast with a declining consumption in other groups in the sample.

## 1. Introduction

Globally, alcohol consumption is among the leading risk factors for premature death and loss of healthy life years [1]. In addition, alcohol consumption is an important risk factor for a range of social problems, including violent crime [2], family disruption, child neglect [3], and work-related problems [4]; the individual risk of health and social problems increases with increasing consumption [2,4,5]. At the population level, an increase in total alcohol consumption is generally accompanied by an increase in the prevalence of heavy drinking [6,7]; therefore, an increase in total consumption is typically accompanied by increased population rates of alcohol-related health and social harms [8], and vice versa (i.e., reduced harm rates accompany a decrease in total consumption). Implicitly, this leads us to the expectation that when the total consumption remains stable, so does the prevalence of heavy drinking and related harm rates. However, this relationship seems not necessarily to be the case always, and a recent study from Norway [9] found increased rates of heavy drinking despite a small decrease in total consumption during the first months of the COVID-19 pandemic. In this study, we explore whether this could also apply to other European countries.

During the first phase of the COVID-19 pandemic, numerous studies showed that a large proportion of current drinkers reported they had changed their consumption, either by drinking less or by drinking more; e.g., [10,11,12,13,14,15,16,17,18,19,20,21,22,23,24]. In many of these studies [10,11,12,14,15,18,19,20,21,22], the proportion reporting a decrease in alcohol consumption was larger than the proportion reporting an increase in consumption. This tendency has been interpreted as an indication of a decrease in overall consumption [23]. This is indeed one possible explanation of the findings, which could be attributed to reduced affordability and availability of alcohol in addition to fewer opportunities for social occasions to drink during the pandemic [25].

A few studies have also found that individual changes in consumption were contingent on the pre-pandemic drinking level; i.e., those with initial low consumption tended to report reduced drinking during the pandemic, whereas the group with the initial highest consumption tended to report increased drinking [21,22]. These findings may suggest two preliminary conclusions; (i) that those drinking at heavy levels changed their consumption differently from other drinkers, and (ii) that despite a preponderance of people reporting reduced drinking, the overall consumption levels—in these survey samples—did not necessarily decrease. For example, the above-mentioned study from Norway [9] found that during the first phase of the COVID-19 pandemic (i.e., March to May 2020), the estimated overall change in alcohol consumption was small. However, among drinkers in the upper 10% of initial consumption (i.e., in the upper 10% of drinkers before or at the start of the pandemic situation), there was an increase in consumption, pushing a number of these drinkers above the high-risk threshold; hence, the estimated prevalence of high-risk drinkers seemed to increase during the first phase of the pandemic. In line with this, studies that examined distributional changes in alcohol consumption at the aggregate level found the prevalence of people reporting heavy drinking to have increased during the pandemic [26,27]. However, to the best of our knowledge, no other studies have examined whether such a pattern (of heavy drinkers increasing their consumption) has emerged across different countries. The findings from the Norwegian study [9] suggests a simple pattern of positive correlation between initial consumption levels and subsequent increases in consumption during the pandemic. However, other European countries had experienced more intrusive social restrictions, such as curfews and stay-at-home-orders, than Norway had (Table 1). Such restrictions may impact consumption in various ways: reduced social contact and fewer opportunities for social drinking as well as diminished incomes may reduce consumption for some, whereas, for others, greater alcohol consumption may result from increased stress or boredom, or from improved possibilities to drink heavily without this being noticed at work [25]. Thus, the impact of the pre-pandemic consumption level on the change in alcohol consumption may also differ by country. Consistent with this reasoning, a pan-European study found an average decrease in overall consumption in 19 countries, yet not in Ireland (no change) nor the UK (increase), and the authors argued that differential exposure to pandemic-related stress could be one possible explanation for the country differences observed [23]. It is therefore of interest to explore whether changes in alcohol consumption during the pandemic follow a similar pattern across countries with differing pandemic-related responses, drinking cultures and societal factors, or whether more complex associations emerge.

In this study, we explored possible inter-country differences with regard to (i) whether individual changes in alcohol consumption during the first months of the COVID-19 pandemic were related to one’s initial consumption level; (ii) whether these individual changes in consumption translated into possible changes in the prevalence of heavy drinking.

## 2. Material and Methods

### 2.1. Samples and Data Collections

We employed data from the European Alcohol Use and COVID-19 survey (ESAC), an online survey targeted at adults aged 18 years or older which captured changes in alcohol consumption during the first months of the COVID-19 pandemic [28]. The online cross-sectional survey was available in 20 languages and took place in 21 European countries using non-probabilistic convenience sampling and by distributing the survey mainly via social media, mailing lists and press releases. All study materials including collected survey data are available online [29]. For the present study, we employed data from the eight countries which had the largest number of respondents: Czechia (n = 1555), Denmark (n = 2566), Finland (n = 3800), Germany (n = 1659), Norway (n = 17,092), Poland (n = 1148), Spain (n = 3139), and the UK (n = 962). Participation in the survey was voluntary and anonymous, and the survey was approved by the Data Protection Office of the Technische Universität Dresden with regards to the EU General Data Protection Regulation 2016/679. Recruitment of ESAC study participants lasted from 24 April to 22 July 2020 [23], but recruitment period varied by country (country-level information is provided here: [29]). Among the eight countries included in this study, recruitment started by the end of April 2020 in seven countries, whereas in Finland, recruitment started on 14 May 2020. Recruitment ended 30 June or 1 July in all countries except Germany (where it ended on 22 July).

We conducted web-searches to obtain information about type and timing of lockdown measures of relevance for alcohol consumption, including restrictions on on-premise and off-premise alcohol sales and restrictions on social gatherings in private homes, for each of the eight countries during the period of March through June 2020 (see Supplementary Table S1B).

### 2.2. Measures

#### 2.2.1. Determining the Level of Initial Consumption

Respondents were asked about their alcohol consumption in the past 12 months (henceforth referred to as initial alcohol consumption), using the AUDIT-C questionnaire [30]. For this study, we applied all three items in the main analyses (frequency of drinking, quantity of alcohol consumed per occasion, and frequency of 6+ units) (see Table S1A for response options). Standard units of alcohol were described per beverage type in terms of nationally defined standard drinks and corresponded to approximately 10/11 g of pure alcohol, except for Czechia (16 g) and the UK (8 g) (see [23] for details). Thus, to obtain comparable measures of consumption across countries, amounts were multiplied with 1.5 in Czechia and 0.8 in the UK. Initial alcohol consumption was estimated as follows: first, the product of drinking frequency in the past 12 months and usual quantity per occasion (see Table S1A for recoding of variables) was divided by 52 (i.e., number of weeks/year) to obtain number of alcohol units consumed per week. For those reporting a usual quantity per occasion to be less than 6 units, we estimated the volume from occasions when 6+ units were consumed (assuming an average of 7.5 units per 6+ drinking occasion), which was added to the volume described above.

#### 2.2.2. Quantifying Changes in Alcohol Consumption in the Course of the Pandemic

Respondents who reported any past-year alcohol consumption (henceforth termed current drinkers) were asked about perceived changes during the past month in (i) drinking frequency, (ii) usual quantity per occasion, and (iii) frequency of drinking 6+ units per occasion (i.e., heavy episodic drinking, HED). All three questions had the following five response options: ‘much less (often)’, ‘slightly less (often)’, ‘no change’, ‘slightly more (often)’ and ‘much more (often)’. These responses took on the values −2, −1, 0, +1 and +2, respectively, and we constructed a simple change index: a sum-score of the three variables divided by three.

Moreover, in line with the previous study from Norway [9], we estimated change in volume of alcohol consumption during the COVID-19 pandemic. Following the application of Weber–Fechner’s law in psychology to alcohol consumption [7], the calculation was based on the assumption that a given description of the quantity of change in consumption (‘much less/more’, ‘slightly less/more’) will be proportional to the initial consumption level. For example, a person consuming 20 L per year will perceive an increase of 5 L equivalently to an increase of 1 L by a person who drinks 4 L per year [7]. Thus, we applied a model of quantifiable relative changes (see Table S1A for detailed description), corresponding to Model 2 in the previous study from Norway [9]. Here, we assumed that ‘much more’ represented an increase of +50% and ‘much less’ represented a decrease of −33%, which reflects a symmetrical relative change (e.g., a 50% increase from 6 gives 9, and a 33% reduction from 9 gives 6). ‘Slightly more’ and ‘slightly less’ corresponded to +10% and −9%, respectively. The magnitude of the relative change assumed in this model is likely to be conservative, considering the substantial individual flux in alcohol consumption from one year to the next, as reported from various countries including the Nordic countries and the USA [31]. From these quantifications of the three variables of changes in drinking behavior, we estimated consumption during the pandemic, and the volume estimate was calculated as explained above. From the estimated initial consumption and the estimated alcohol consumption during the pandemic, we calculated change in alcohol consumption from the initial level to that during the pandemic (in number of alcohol units per week). Missing data/invalid responses on each variable ranged from 0.8% to 1.4%.

#### 2.2.3. Setting a Cut-Off for Heavy Drinking

There is no single way of operationalizing heavy drinking. The overall risk of health harms from drinking increases monotonously with increasing consumption [5], and hence various thresholds for heavy drinking are used [32]. In this study, we applied a threshold of 28 units per week. At this level, all-cause mortality risk is increased by over 20% [33].

### 2.3. Statistical Analyses

The analyses were explorative. The sample distribution according to gender, age and educational level in the ESAC surveys deviated from that in the actual adult populations in the eight countries [34], and hence sample weights accounting for the skewed sample distribution of gender, age and educational level were applied to the analyses (see [35] for a detailed description). Initial alcohol consumption was categorized by employing the 25th, 50th, 75th, and 90th percentiles of the distribution as cut-offs within each country. Changes in alcohol consumption (change index score and estimated change in units per week) by initial consumption categories were described graphically, and differences between those above the 90th percentile and other drinkers were tested with an F-test. Differences in prevalence of heavy drinking were tested by comparing the two proportions that determine changes in heavy drinking prevalence: (a) the share of all respondents who were below the heavy drinking threshold initially but passed the threshold during the pandemic (new heavy drinkers) to (b) the share of all respondents who drank above the heavy drinking threshold initially, but decreased their drinking to below that threshold during the pandemic (former heavy drinkers). These proportions were compared via means of two-tailed proportion tests.

In sensitivity analyses we also employed another approach to measuring consumption by calculating initial volume from drinking frequency and usual quantity only. Changes in alcohol consumption were calculated as described above, excluding the change in 6+ frequency. Moreover, we also employed an alternative set of quantifications of reported changes in drinking behavior (see Supplementary Table S1A).

## 3. Results

The analytical samples comprised current drinkers with valid answers on the three AUDIT-C items and the three variables on perceived changes in drinking (see Table 1). The most important societal-level measures of restrictions which were introduced before the data collection are also presented by country. In all countries, national pandemic-related restrictions included closed on-premise sales of alcohol, and there were no restrictions on off-premise alcohol sales. However, restrictions on social gatherings in private homes varied between countries, and in particular, four of the countries; Czechia, Germany, Spain and the UK, experienced more severe restrictions with regard to this measure.

For each country, Table 2 presents means for the initial consumption, the estimated change in volume of consumption and the change index score. The latter two are presented for all and by initial consumption level, with the upper 10% drinkers distinguished from the others. A clear pattern emerged from all countries: the estimated change in consumption in units per week was statistically significantly higher among the upper 10% of the drinkers than among the rest. In all countries, except for the UK, the estimated change in consumption among all drinkers was quite small. Thus, also relative to initial consumption, the increase in consumption was clearly higher in the UK than in the other countries. Moreover, we can observe from Table 2 that at the country level, the estimated change in consumption and the change index score tended to be higher with increasing initial consumption level.

**Figure 1:**
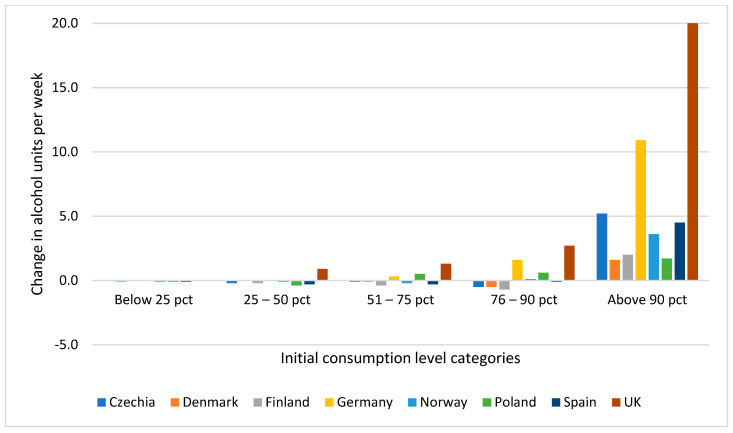
Change in estimated volume of consumption by initial consumption level and country. Note: Initial consumption level categories are based on percentiles of the distribution in each country.

We plotted estimated change in consumption volume by level of initial consumption and country (Figure 1). This graph shows that in seven countries (Czechia, Denmark, Finland, Germany, Norway, Poland and Spain) and among those at initial consumption levels below the 90th percentile, there was little change in consumption from initial to during the pandemic. Additionally, there was no clear patterning in the direction of change. In contrast, among those with the highest initial consumption (above the 90th percentile), there was a marked increase in consumption. In the UK, with the highest average initial consumption of the countries studied, an increase in consumption from initial level to the period during the pandemic was observed also among consumer groups below the 90th percentile (Figure 1).

Finally, we explored whether the proportion of heavy drinkers had increased from before to during the pandemic. For each country, we examined the proportion exceeding 28+ units per week initially and during the first months of the pandemic. The proportion of heavy drinkers increased during the pandemic among survey respondents in seven of the eight countries (Table 3).

We conducted two sets of sensitivity analyses. Findings from the first set are reported in Tables S2A and S3A and Figure S1A, which parallel Table 2 and Table 3 and Figure 1 in the main analysis, respectively. Here, we applied measures which excluded HED in the calculations of initial alcohol consumption and change in alcohol consumption during the pandemic (i.e., measures based on frequency and usual quantity) (Table S1A). These analyses showed similar findings as those described for the main analyses: while the upper 10% of drinkers increased their consumption, the remainder had changed their consumption to a very small extent, or—as in Germany or the UK—had increased their consumption to a lesser extent (Table S2A). This is further illustrated in Figure S1A, showing that in seven countries, estimated change in consumption was small and varied little by consumption level below the 90th percentile, whereas in the UK an increase in consumption was observed at all consumption levels above the median. With regard to changes in the prevalence of heavy drinkers, these sensitivity analyses showed that in all eight countries there was a statistically significant increase in the proportion of heavy drinkers (Table S3A).

Findings from the second sensitivity analysis are reported in Tables S2B and S3B and Figure S1B, which parallel Table 2 and Table 3 and Figure 1 in the main analysis. Here, we applied measures that included HED but assumed a larger relative change in drinking frequency, usual quantity per occasion and consumption from HED occasions (Table S1A). These analyses yielded similar findings as those in the main analyses: a substantial increase in consumption among the upper 10% of drinkers initially (Table S2B), and an increase in the proportion of heavy drinkers in seven of the eight countries (Table S3B). The estimated change in consumption by initial consumption levels followed the same pattern as in the main analysis (Figure S1B).

## 4. Discussion

We examined changes in alcohol consumption during the first months of the COVID-19 pandemic (i.e., April through June 2020) and found that across eight European countries, despite somewhat differing pandemic-related social restrictions, individual changes in alcohol consumption followed a similar pattern. Those drinkers with the highest initial or pre-pandemic consumption levels had increased their consumption during the pandemic, whereas small average changes in consumption volume were found among other drinkers. The findings also suggest that the proportion of drinkers exceeding 28 units of alcohol per week increased from before to during the first months of the pandemic.

Other studies have examined whether changes in alcohol consumption during the pandemic differed by initial consumption level. In addition to previous reports employing ESAC project data from Germany [22] and Norway [9], results from a number of studies employing other data sets and methods [21,36,37,38] corroborate our findings, showing that increased drinking was associated with higher initial consumption levels. In contrast, some studies have found that an increase in consumption was associated with a lower initial consumption level [10,39]. In line with our findings, previous studies have also reported indications that heavy drinking has increased during the pandemic [18,38]. For instance, in England, the proportion of heavy drinkers (scoring 5+ on AUDIT-C) increased from 25% pre-pandemic to 38% during the April 2020 lockdown [26]. Notably, the mixed findings in the literature reviewed above may well reflect various methodological differences. The novelty of our study pertains to the exploration of possible differences in changes in consumption and heavy drinking, by employing identical methodology across a selection of countries.

Our study suggests—without implying causality—that the COVID-19 pandemic has affected those who drink at high levels differently to other drinkers, with an overall increase in alcohol consumption among the heavier drinkers and a very small change (in either direction) in alcohol consumption among the remainder. Following regression to the mean, we would expect to see the opposite trends in drinking level changes. Thus, this observed polarization of alcohol consumption may be a phenomenon associated with a specific type of public health crisis.

While there was—in most countries—only a small change in mean consumption, the observed polarization of alcohol consumption appears to deviate from our general understanding of the ‘total consumption model’ and the idea of a close relationship between changes in mean and dispersion of the consumption distribution [8]. However, it is conceivable that certain pandemic-related factors impacting consumption (e.g., social distancing or isolation and less informal social control when working from home) differ substantially or exert a different impact on heavy drinkers compared to other drinkers, whereas other factors that usually impact consumption (e.g., affordability and physical availability) affect all consumer groups fairly equally. In line with this reasoning, a study from the US [40] found that hazardous alcohol use and possible dependence increased month-by-month for those under lockdowns compared to those not under restrictions, which may suggest that an increase in heavy drinking was a response to being in lockdown.

With regard to possible inter-country differences, our findings suggest that overall, there were no obvious or distinct differences between countries regarding the polarization of changes in alcohol consumption during the first phase of the pandemic, despite some differences in national restrictions on social gatherings in private homes and stay at home orders. While these were more severe in Czechia, Germany, Spain and the UK, and may thus have impacted social availability of alcohol, the polarization of changes in these four countries did not emerge as systematically different from those of the other countries. However, the relative size of changes in consumption did vary by country: in particular, in the UK we observed a higher relative increase in consumption. The markedly higher initial consumption in the UK sample compared with the other countries, may result from a sampling bias. The proportion of heavy drinkers was higher in this study compared to a previous study based on a representative survey in England [41], suggesting that heavier drinkers may have been over-represented in the UK sample.

This study extends many previous studies on changes in alcohol use during the COVID-19 pandemic [10,11,12,13,14,15,16,17,18,19,20,21,22,23] by providing quantifications of qualitative assessments of changes in consumptions, and it broadens the scope of the previous Norwegian study [9] by demonstrating that the same pattern of change in alcohol consumption is found in eight European countries. Nevertheless, some potential limitations warrant attention. This study is based on data from a web-survey of convenience samples, employing various sampling methods [23], and although data were weighted, there may be biases in the consumption distribution [42] and in reported changes in consumption during the pandemic for each country and across countries. Underreporting in survey research on alcohol consumption is well-known [38]. In addition, the format of the ESAC survey failed to identify people who had been abstinent in the year before the pandemic, but who began drinking during the pandemic, which may have led to an underestimation of an increase in consumption. The estimations of changes in alcohol consumption in the current study are based on theory-driven assumptions [4], and the results are only as robust as these assumptions. Yet, the sensitivity analyses and the similar findings with regard to change in volume of consumption and change index score, suggest that the observed pattern of polarization is robust. The sample size and representativeness did not allow analysis by gender, which might be a relevant factor given the different contexts in which men and women drink in different countries. Country-specific socioeconomic differences in digital skills and possession of Internet-connected devices [43] may have also prevented some of those at heavy drinking levels access to the website-based survey, with impacts on the representativeness of the samples. Finally, our assessment of national pandemic-related restrictions was crude, and we could not account for or standardize all the different characteristics of all relevant national and regional restrictions and interactions with existing policy, which means that drawing conclusions about the mechanism of this change is not possible.

The implications of our findings pertain in particular to the likely increase in the prevalence of heavy drinkers, who are more vulnerable than others to infectious diseases, including COVID-19 [38]. Moreover, an increase in the number of heavy drinkers implies an additional burden on a variety of health care services during an already challenging period, as heavy drinkers are at high risk of acute injuries and substantial risk of new—or complications of existing—somatic or mental health problems [1,38,42]. There is also some indication that heavy drinkers (especially when drinking) are less able or likely to comply with social distancing recommendations [44,45], or vaccination schedules, increasing the COVID-19-risk for this vulnerable group. Further research into the possible effects of the pandemic on the distribution of alcohol consumption and heavy drinking is needed, in order to plan pandemic responses more effectively. Similarly, there is a need to provide health care and possible treatment to those at pre-clinical but high levels who are at risk of increasing their drinking during the pandemic, with negative health consequences.

## 5. Conclusions

In conclusion, quantifications of reported changes in alcohol consumption during the pandemic suggest similar patterns of polarization across eight European countries; those with initial drinking levels in the upper 10% increased their consumption substantially and the prevalence of heavy drinkers increased, whereas consumption changed very little among the remaining majority of drinkers.

## Figures and Tables

**Table 1:** Analytic sample size, proportion of drinkers in total sample and main national pandemic-related restrictions relevant for alcohol consumption in the first phase of the pandemic, by country.

	Analytic Sample Un-Weighted	Proportion Drinkers in Total Sample % (n)	Main National Restrictions Due to Pandemic in Month Prior to Survey
Czechia	1389	92.2 (1434)	From 12 March to 17 May, nationwide curfew, closed on-premise alcohol sales. From 15 March to 24 April, hard restrictions on social gathering inside and outside private homes.
Denmark	2369	95.0 (2437)	From 11 March to 18 May, closed on-premise alcohol sales. Minor restrictions on social gatherings in private homes.
Finland	3389	91.3 (3471)	From 16 March to 31 May, closed on-premise alcohol sales. Minor restrictions on social gatherings in private homes.
Germany	1470	91.4 (1517)	From 22 March closed on-premise alcohol sales, re-opening from mid-May. Substantial restrictions on social gatherings inside and outside private homes. Regulations and timing partly varied between the federal states.
Norway	15,220	90.8 (15,520)	From 12 March to 1 June, closed on-premise alcohol sales. Minor restrictions on social gatherings in private homes.
Poland	1065	94.9 (1090)	From 13 March to 18 May, closed on-premise alcohol sales. From 24 March to 25 June, minor restrictions on social gatherings, also in private homes.
Spain	2660	88.3 (2773)	From 14 March to 21 June (with some regional variations from 28 April), closed on-premise alcohol sales. Social restrictions including stay-at-home order for all non-essential workers, and a ban on gatherings inside and outside, with some exceptions.
UK	886	95.6 (920)	From 23 March to 10 May, lock-down. Closed on-premise sales until 4 July. Substantial restrictions on social gatherings also in private homes.

Note: In all countries, alcohol was available in food stores/grocery shops and in the alcohol monopoly outlets in Finland and Norway. See Table S1B for overview of links to sources of information regarding pandemic-related restrictions.

**Table 2:** Initial consumption, change in alcohol consumption for all and by initial consumption level (below vs. above 90th percentile), and change index score for all and by initial consumption level (below vs. above 90th percentile), by country.

	Alcohol Consumption in Units per Week, Mean (95% CI)					Change Index Score, Mean (95% CI)			
	Initial Level	Change from Initial Level to Pandemic				Change from Initial Level to Pandemic			
		All	Below 90 pct	Above 90 pct	*p*	All	Below 90 pct	Above 90 pct	*p* *
Czechia	12.6 (11.7, 13.6)	0.6 (0.2, 1.0)	−0.2 (−0.3, 0.0)	5.2 (2.7, 7.7)	<0.001	−0.17 (−0.22, −0.11)	−0.24 (−0.30, −0.19)	0.30 (0.15, 0.37)	<0.001
Denmark	8.0 (7.6, 8.4)	0.1 (−0.1, 0.3)	−0.2 (−0.2, −0.1)	1.6 (0.5, 2.7)	<0.001	−0.20 (−0.24, −0.17)	−0.25 (−0.28, −0.21)	0.03 (−0.08, 0.08)	<0.001
Finland	7.7 (7.3, 8.0)	0.0 (−0.2, 0.3)	−0.3 (−0.4, −0.3)	2.0 (0.6, 3.3)	<0.001	−0.37 (−0.40, −0.34)	−0.45 (−0.48, −0.41)	0.02 (−0.08, 0.07)	<0.001
Germany	9.8 (9.1, 10.5)	1.5 (1.1, 1.9)	0.3 (0.1, 0.5)	10.9 (7.8, 14.0)	<0.001	−0.06 (−0.11, −0.01)	−0.13 (−0.18, −0.08)	0.51 (0.34, 0.60)	<0.001
Norway	6.8 (6.6, 6.9)	0.4 (0.3, 0.5)	−0.1 (−0.1, −0.1)	3.6 (3.0, 4.2)	<0.001	−0.27 (−0.28, −0.25)	−0.33 (−0.35, −0.32)	0.19 (0.14, 0.21)	<0.001
Poland	9.3 (8.5, 10.1)	0.22 (−0.1, 0.5)	0.1 (−0.1, 0.2)	1.7 (−0.9, 4.3)	<0.001	−0.23 (−0.29, −0.17)	−0.28 (−0.34, −0.21)	0.21 (−0.03, 0.33)	<0.001
Spain	8.0 (7.5, 8.5)	0.48 (0.2, 0.8)	−0.2 (−0.3, −0.1)	4.5 (2.4, 6.6)	<0.001	−0.41 (−0.46, −0.37)	−0.48 (−0.53, −0.43)	0.00 (−0.15, 0.08)	<0.001
UK	16.9 (15.9, 17.9)	4.9 (4.0, 5.8)	1.3 (0.8, 1.8)	20.1 (16.6, 23.7)	<0.001	0.27 (0.19, 0.35)	0.11 (0.03, 0.20)	0.93 (0.74, 1.02)	<0.001

Note: The estimated change in consumption was constructed from questions about changes in drinking frequency, usual amount per occasion and HED frequency and assumed changes relative to initial drinking behavior, and the change was calculated in alcohol units per week. The change index was constructed from questions about changes in drinking frequency, usual amount per occasion and HED frequency, and the score ranged from −1 to +1. * F-test for difference in consumption change/index score change by initial consumption level (below vs. above 90th percentile).

**Table 3:** Estimated proportion of heavy drinkers initially and during the pandemic, by country. Percent (95% CI).

Country	Exceeding 28 Units/Week		
	Initially	during Pandemic	*p* *
Czechia	13.2 (11.4–15.1)	13.7 (11.8–15.6)	0.148
Denmark	3.1 (2.4–3.8)	5.7 (4.8–6.6)	<0.001
Finland	4.1 (3.4–4.7)	5.8 (5.0–6.5)	<0.001
Germany	6.8 (5.5–8.1)	10.2 (8.6–11.7)	<0.001
Norway	3.2 (2.9–3.5)	4.6 (4.3–4.9)	<0.001
Poland	4.4 (3.1–5.7)	7.2 (5.6–8.8)	<0.001
Spain	5.4 (4.5–6.3)	7.5 (6.5–8.5)	<0.001
UK	24.1 (21.3–26.9)	26.0 (23.1–28.9)	0.007

Note: * *p*-value for proportion test.

## Data Availability

The dataset is available in the Figshare repository, https://doi.org/10.6084/m9.figshare.13580693.v1 (accessed on 15 January 2021).

## Supplementary Materials

The following are available online at www.mdpi.com/article/10.3390/ijerph181910547/s1, Table S1A: Overview of how qualitative responses to questions about changes in drinking frequency, usual quantity per occasion and heavy episodic drinking were translated into quantified measures of drinking frequency and usual quantity per occasion and quantity from heavy episodic drinking during the pandemic based on responses to questions on initial consumption (AUDIT-C, questions 1, 2 and 3) in main analyses and sensitivity analyses., Table S1B: Sources for information about pandemic-related social measures, by country., Table S2A: Changes in alcohol use by change indicator (change index and estimated change in volume of consumption) by initial consumption level and country., Table S2B: Changes in alcohol consumption (assuming larger relative changes) by initial consumption level and country., Table S3A: Estimated proportion exceeding 28 units per week initially and during the pandemic, by country., Table S3B: Estimated proportion exceeding 28 units per week initially and during the pandemic, by country. Figure S1A: Change in estimated volume of consumption by initial consumption level and country. Sensitivity analysis: HED excluded from calculations of initial alcohol consumption and quantified change in alcohol consumption., Figure S1B: Change in estimated volume of consumption by initial consumption level and country. Sensitivity analysis: assuming a larger relative quantified change in drinking frequency, usual quantity per occasion and consumption from HED occasions.

## References

[1] Shield K., Manthey J., Rylett M., Probst C., Wettlaufer A., Parry C.D., Rehm J. (2020). National, regional, and global burdens of disease from 2000 to 2016 attributable to alcohol use: A comparative risk assessment study. Lancet Public Health.

[2] Rossow I., Bye E.K., McMurran M. (2013). The problem of alcohol-related violence: An epidemiological and public health perspective. Alcohol-related Violence: Prevention and Treatment.

[3] Laslett A.-M., Giesbrecht N., Bosma L.M. (2017). Alcohol’s harm to others: Evidence and options for community action. Preventing Alcohol-Related Problems.

[4] Anderson P. (2012). Alcohol and the Workplace. Alcohol in the European Union: Consumption, Harm and Policy Approaches.

[5] Griswold M.G., Fullman N., Hawley C., Arian N., Zimsen S.R.M., Tymeson H.D., Venkateswaran V., Tapp A.D., Forouzanfar M.H., Salama J.S. (2018). Alcohol use and burden for 195 countries and territories, 1990–2016: A systematic analysis for the Global Burden of Disease Study 2016. Lancet.

[6] Rossow I., Mäkelä P., Kerr W. (2014). The collectivity of changes in alcohol consumption revisited. Addiction.

[7] Skog O.-J. (1985). The collectivity of drinking cultures: A theory of the distribution of alcohol consumption. Br. J. Addict..

[8] Rossow I., Mäkelä P. (2021). Public health thinking around alcohol-related harm: Why does per capita consumption matter?. J. Stud. Alcohol Drugs.

[9] Rossow I., Bye E.K., Moan I.S., Kilian C., Bramness J.G. (2021). Changes in Alcohol Consumption during the COVID-19 Pandemic—Small Change in Total Consumption, but Increase in Proportion of Heavy Drinkers. Int. J. Environ. Res. Public Health.

[10] Ritter A., Wilkinson C., Vuong T., Kowalski M., Barrett L., Mellor R., Sommerville K. (2020). Distilling Our Changing Relationship with Alcohol during COVID-19.

[11] Callinan S., Smit K., Mojica-Perez Y., D’Aquino S., Moore D., Kuntsche E. (2021). Shifts in alcohol consumption during the COVID-19 pandemic: Early indications from Australia. Addiction.

[12] Tran T.D., Hammarberg K., Kirkman M., Nguyen H.T.M., Fisher J. (2020). Alcohol use and mental health status during the first months of COVID-19 pandemic in Australia. J. Affect. Disord..

[13] Biddle N., Edwards B., Gray M., Sollis K. (2020). Alcohol consumption during the COVID19 period: May 2020. ANU Centre for Social Research and Methods.

[14] Vanderbruggen N., Matthys F., Van Laere S., Zeeuws D., Santermans L., Van den Ameele S., Crunelle C.L. (2020). Self-Reported Alcohol, Tobacco, and Cannabis Use during COVID-19 Lockdown Measures: Results from a Web-Based Survey. Eur. Addict. Res..

[15] Panagiotidis P., Rantis K., Holeva V., Parlapani E., Diakogiannis I. (2020). Changes in Alcohol Use Habits in the General Population, during the COVID-19 Lockdown in Greece. Alcohol Alcohol..

[16] Chodkiewicz J., Talarowska M., Miniszewska J., Nawrocka N., Bilinski P. (2020). Alcohol consumption reported during the COVID-19 pandemic: The initial stage. Int. J. Environ. Res. Public Health.

[17] Koopmann A., Georgioadou E., Kiefer F., Hillemacher T. (2020). Did the general population in Germany drink more alcohol during the COVID-19 pandemic lockdown?. Alcohol Alcohol..

[18] Pollard M.S., Tucker J., Green H.D. (2020). Changes in Adult Alcohol Use and Consequences During the COVID-19 Pandemic in the US. JAMA Netw. Open.

[19] Sallie S.N., Ritou V., Bowden-Jones H., Voon V. (2020). Assessing international alcohol consumption patterns during isolation from the COVID-19 pandemic using an online survey: Highlighting negative emotionality mechanisms. BMJ Open.

[20] Alpers S.E., Skogen J.C., Mæland S., Pallesen S., Rabben Å.K., Lunde L.-H., Fadnes L.T. (2021). Alcohol Consumption during a Pandemic Lockdown Period and Change in Alcohol Consumption Related to Worries and Pandemic Measures. Int. J. Environ. Res. Public Health.

[21] Bramness J.G., Bye E.K., Moan I.S., Rossow I. (2021). Norwegians’ alcohol use during the COVID-19 pandemic: Self-reported changes and motives for change. Eur. Addict. Res..

[22] Manthey J., Kilian C., Schomerus G., Kraus L., Rehm J., Schulte B. (2020). Alkoholkonsum in Deutschland und Europa während der SARS-CoV-2 Pandemie. Sucht.

[23] Kilian C., Rehm J., Allebeck P., Braddick F., Guall A., Bartak M., Bloomfield K., Gil A., Neufeld M., O’Donnell A. (2021). Alcohol consumption during COVID-19 pandemic in Europe: A large-scale cross-sectional study in 21 countries. Addiction.

[24] Mäkelä P., Rossow I., Moan I.S., Bye E.K., Kilian C., Raitasalo K., Allebeck P. (2021). Measuring changes in alcohol use in Finland and Norway during the COVID-19 pandemic: Comparison between data sources. Int. J. Methods Psychiatr. Res..

[25] Rehm J., Kilian C., Ferreira-Borges C., Jernigan D., Monteiro M., Parry C., Sanchez Z.M., Manthey J. (2020). Alcohol use in times of the COVID 19: Implications for monitoring and policy. Drug Alcohol Rev..

[26] Jackson S.E., Garnett C., Shahab L., Oldham M., Brown J. (2021). Association of the COVID-19 lockdown with smoking, drinking and attempts to quit in England: An analysis of 2019–20 data. Addiction.

[27] Daly M., Robinson E. (2021). High-risk drinking in midlife before versus during the COVID-19 crisis: Longitudinal evidence from the United Kingdom. Am. J. Prev. Med..

[28] (2020). Covid19 and Alcohol. https://www.covid19-and-alcohol.eu/.

[29] Kilian C. (2020). Dissemination Strategies 1.2. Figshare. https://figshare.com/articles/dataset/Dissemination_strategies_1_X/12738728/3.

[30] Higgins-Biddle J.C., Babor T.F. (2018). A review of the Alcohol Use Disorders Identification Test (AUDIT), AUDIT-C, and USAUDIT for screening in the United States: Past issues and future directions. Am. J. Drug Alcohol Abus..

[31] Skog O.-J., Rossow I. (2006). Flux and stability: Individual fluctuations, regression towards the mean and collective changes in alcohol consumption. Addiction.

[32] Dawson D.A. (2011). Defining risk drinking. Alcohol Res. Health.

[33] Wood A.M., Kaptoge S., Butterworth A.S., Willeit P., Warnakula S., Bolton T., Paige E., Paul D.S., Sweeting M., Burgess S. (2018). Risk thresholds for alcohol consumption: Combined analysis of individual-participant data for 599 912 current drinkers in 83 prospective studies. Lancet.

[34] EUROSTAT (2020). Dataset: Population by Educational Attainment Level, Sex and Age (1000). https://appsso.eurostat.ec.europa.eu/nui/show.do?dataset=edat_lfs_9901&lang=en.

[35] Kilian C. (2020). Survey Weights. Figshare. Dataset. https://figshare.com/articles/Survey_weights/12739469/1.

[36] Neill E., Meyer D., Toh W.L., van Rheenen T.E., Phillipou A., Tan E.J., Rossell S.L. (2020). Alcohol use in Australia during the early days of the COVID-19 pandemic: Initial results from the COLLATE project. Psychiatry Clin. Neurosci..

[37] Sidor A., Rzymski P. (2020). Dietary choices and habits during COVID-19 lockdown: Experience from Poland. Nutrients.

[38] Murthy P., Narasimha V.L. (2021). Effects of the COVID-19 pandemic and lockdown on alcohol use disorders and complications. Curr. Opin. Psychiatry.

[39] Pabst A., Bollen Z., Creupelandt C., Fontesse S., Orban T., de Duve M., Pinon N., Maurage P. (2021). Alcohol consumption changes during the first COVID-19 lockdown: An online population survey in a convenience sample of French-speaking Belgian residents. Psychiatry Res..

[40] Killgore W.D.S., Cloonan S.A., Taylor E.C., Lucas D.A., Dailey N.S. (2021). Alcohol dependence during COVID-19 lockdowns. Psychiatry Res..

[41] Lewer D., Meier P., Beard E., Boniface S., Kaner E. (2016). Unravelling the alcohol harm paradox: A population-based study of social gradients across very heavy drinking thresholds. BMC Public Health.

[42] Sherk A., Stockwell T., Rehm J., Dorocicz J., Shield K.D. (2017). A Comprehensive Guide to the Estimation of Alcohol-Attributable Morbidity and Mortality.

[43] Van Deursen A.J., Van Dijk J.A. (2019). The first-level digital divide shifts from inequalities in physical access to inequalities in material access. New Media Soc..

[44] Gurrieri L., Fairbairn C.E., Sayette M.A., Bosch N. (2021). Alcohol narrows physical distance between strangers. Natl. Acad. Sci..

[45] Suffoletto B., Ram N., Chung T. (2020). In-person contacts and their relationship with alcohol consumption among young adults with hazardous drinking during a pandemic. J. Adolesc. Health.

