# Supplementary material for: Changes in Alcohol Consumption during the COVID-19 Pandemic Are Dependent on Initial Consumption Level: Findings from Eight European Countries
Source: Int J Environ Res Public Health. 2021 Oct 8;18(19):10547. doi: 10.3390/ijerph181910547 (PMC8508389; doi:10.3390/ijerph181910547)
Supplement: Supplementary file 1 [file ijerph-18-10547-s001.zip › ijerph-1399417-supplementary.pdf]

International Journal of
Environmental Research and Public Health

MDPI

Supplementary material for Ms: Changes in alcohol consumption during the COVID-19 pandemic are dependent on initial consumption level: Findings from eight European countries.

Table S1A. Overview of how qualitative responses to questions about changes in drinking frequency, usual quantity per occasion and heavy episodic drinking were translated into quantified measures of drinking frequency and usual quantity per occasion and quantity from heavy episodic drinking during the pandemic based on responses to questions on initial consumption (AUDIT-C, questions 1, 2 and 3) in main analyses and sensitivity analyses .

		Main analyses					Sensitivity analyses I					Sensitivity analyses II				
		Much less	A little less	No change	A little more	Much more	Much less	A little less	No change	A little more	Much more	Much less	A little less	No change	A little more	Much more
**AUDIT-C question 1**																
Quantified change		-33%	-9%	0	+10%	+50%	-33%	-9%	0	+10%	+50%	-50%	-13%	0	+15%	+100%
Monthly or less	= 6[a]	4	5.5	6	6.6	9	4	5.5	6	6.6	9	3	5.2	6	6.9	12
2-4 times a month	=40[a]	26	36	40	44	60	26	36	40	44	60	20	35	40	46	80
2-3 times/week	=120[a]	79	109	120	132	180	79	109	120	132	180	60	104	120	138	240
4 + times a week	=250[a]	165	228	250	275	365	165	228	250	275	365	125	218	250	288	365
**AUDIT-C question 2**																
Quantified change		-33%	-9%	0	+10%	+50%	-33%	-9%	0	+10%	+50%	-50%	-13%	0	+15%	+100%
1 or 2	=1.5[b]	1.0	1.4	1.5	1.7	2.3	1.0	1.4	1.5	1.7	2.3	0.7	1.3	1.5	1.7	3.0
3 or 4	=3.5[b]	2.3	3.2	3.5	3.9	5.3	2.3	3.2	3.5	3.9	5.3	1.8	3.0	3.5	4.0	7.0
5 or 6	=5.5[b]	3.6	5.0	5.5	6.1	8.3	3.6	5.0	5.5	6.1	8.3	2.8	4.8	5.5	6.3	11.0
7-9	=8.0[b]	5.3	7.3	8.0	8.8	12.0	5.3	7.3	8.0	8.8	12.0	4.0	7.0	8.0	9.2	16.0
10 or more	=12.0[b]	7.9	10.9	12.0	13.2	18.0	7.9	10.9	12.0	13.2	18.0	6.0	10.4	12.0	13.8	24.0
**AUDIT-C question 3**																
Quantified change		-33%	-9%	0	+10%	+50%						-50%	-13%	0	+15%	+100%
Less than monthly	=6[c]	4	5.5	6	6.6	9						3	5.2	6	6.9	12
Monthly	=15[c]	10	13	15	17	22.5						7.5	13	15	17	30
Weekly	=50[c]	35	45	50	55	75						25	45	50	55	100

Daily/almost daily	=300 [c]	200	270	300	330	365		150	270	300	330	365

[a] Given value for number of drinking occasions in the past 12 months;
[b] Given value for usual number of alcohol units per drinking occasion;
[c] Given value for number of heavy drinking occasions in the past 12 months

Table S1B. Sources for information about pandemic-related social measures, by country

	Links to information sources
Czechia	https://www.vlada.cz/en/media-centrum/aktualne/measures-adopted-by-the-czech-government-against-coronavirus-180545/<br>https://en.wikipedia.org/wiki/COVID-19_pandemic_in_the_Czech_Republic
Denmark	https://www.garda.com/crisis24/news-alerts/324561/denmark-additional-domestic-restrictions-implemented-march-18-update-5<br>https://www.garda.com/crisis24/news-alerts/325961/denmark-lockdown-extended-until-april-13-due-to-covid-19-update-6<br>https://www.garda.com/crisis24/news-alerts/325961/denmark-lockdown-extended-until-april-13-due-to-covid-19-update-6<br>https://www.garda.com/crisis24/news-alerts/334566/denmark-health-ministry-to-lift-restrictions-on-public-gatherings-from-may-10-update-10<br>https://www.reuters.com/article/us-health-coronavirus-denmark-idUSKBN22J2W2
Finland	https://en.wikipedia.org/wiki/COVID-19_pandemic_in_Finland<br>https://blog.petrieflom.law.harvard.edu/2020/05/14/finland-global-responses-covid19/<br>https://www.reuters.com/article/us-health-coronavirus-finland-idUSKBN22G2AB
Germany	https://www.bundesregierung.de/breg-de/leichte-sprache/22-maerz-2020-regeln-zum-corona-virus-1733310.<br>https://en.wikipedia.org/wiki/COVID-19_pandemic_in_Germany#March_2020<br>https://www.statista.com/statistics/1105090/coronavirus-restaurant-visitation-impact-german/
Norway	https://www.regjeringen.no/en/topics/koronavirus-covid-19/timeline-for-news-from-norwegian-ministries-about-the-coronavirus-disease-covid-19/id2692402/
Poland	https://en.wikipedia.org/wiki/COVID-19_pandemic_in_Poland<br>https://study.gov.pl/news/easing-covid-19-restrictions-poland-may-1-what-will-be-changed

Spain	https://en.wikipedia.org/wiki/COVID-19_pandemic_in_Spain<br>https://home.kpmg/xx/en/home/insights/2020/04/spain-government-and-institution-measures-in-response-to-covid.html
UK	https://en.wikipedia.org/wiki/Timeline_of_the_COVID-19_pandemic_in_the_United_Kingdom_(January%E2%80%93June_2020)<br>https://en.wikipedia.org/wiki/COVID-19_pandemic_in_the_United_Kingdom

Table S2A. Changes in alcohol use by change indicator (change index and estimated change in volume of consumption) by initial consumption level and country.

	Alcohol consumption in units per week, mean (95% CI)					Change index score, mean (95% CI)			
		Change from initial level to pandemic				Change from initial level to pandemic			
	Initial level	All	Below 90 pct	Above 90 pct	p*	All	Below 90 pct	Above 90 pct	p *
Czechia	8.3	0.9	0.4	6.2	<.001	-0.11	-0.15	0.28	<.001
Denmark	6.5	0.3	0.0	2.4	<.001	-0.12	-0.14	0.01	.013
Finland	6.3	0.2	-0.1	4.3	<.001	-0.33	-0.37	0.10	<.001
Germany	6.9	1.6	0.6	12.7	<.001	0.05	0.0	0.68	<.001
Norway	5.5	0.5	0.0	5.8	<.001	-0.19	-0.24	0.32	<.001
Poland	6.8	0.4	0.2	2.6	<.001	-0.15	-0.18	0.28	<.001
Spain	6.0	0.7	-0.1	7.5	<.001	-0.34	-0.41	0.29	<.001
UK	14.3	4.9	2.8	19.6	<.001	0.34	0.27	0.88	<.001

Note: Sensitivity analysis: HED excluded from calculations of initial alcohol consumption and quantified change in alcohol consumption. The estimated change in consumption was constructed from questions about changes in drinking frequency and in usual amount per occasion and assumed changes relative to initial drinking behaviour, and the change was calculated in alcohol units per week. The change index was constructed from questions about changes in drinking frequency and in usual amount per occasion, and the score ranged from -1 to +1. *F-test for difference in consumption change/index score change by initial consumption level (below vs above 90th percentile).

Table S2B. Changes in alcohol consumption (assuming larger relative changes) by initial consumption level and country.

	Change estimated alcohol consumption			
	All drinkers	Below 90 pct	Above 90 pct	P*
Czechia	1.3	0.5	9.4	<.001
Denmark	0.3	-0.1	3.6	<.001
Finland	0.3	-0.5	7.9	<.001
Germany	2.6	0.8	21.6	<.001
Norway	0.8	-0.1	9.7	<.001
Poland	0.5	0.2	2.9	<.001
Spain	1.1	-0.2	12.8	<.001
UK	8.5	5.2	30.8	<.001

Note: Sensitivity analysis: assuming a larger relative quantified change in drinking frequency, usual quantity per occasion and consumption from HED occasions. The estimated change in consumption was constructed from questions about changes in drinking frequency, in usual amount per occasion and HED frequency and assumed changes relative to initial drinking behaviour, and the change was calculated in alcohol units per week. *F-test for difference in consumption change by initial consumption level (below vs above 90th percentile).

Table S3A. Estimated proportion exceeding 28 units per week initially and during the pandemic, by country.

	Initially	During pandemic	P*
Czechia	4.8	8.7	<.001
Denmark	1.5	4.1	<.001
Finland	2.7	4.6	<.001
Germany	2.4	6.2	<.001
Norway	1.8	3.7	<.001
Poland	1.4	4.8	<.001
Spain	2.1	5.0	<.001
UK	15.9	21.1	<.001

Note: Sensitivity analysis: HED excluded from calculations of initial alcohol consumption and quantified change in alcohol consumption. *p-value for proportion test

Table S3B. Estimated proportion exceeding 28 units per week initially and during the pandemic, by country.

Country	Initially	During pandemic	P*
Czechia	13.2	13.6	.820
Denmark	3.1	6.4	<.001
Finland	4.1	6.3	<.001
Germany	6.8	11.5	<.001
Norway	3.2	5.1	<.001
Poland	4.4	8.2	<.001
Spain	5.4	8.4	<.001
UK	24.1	27.9	<.001

Note: Sensitivity analysis: assuming a larger relative quantified change in drinking frequency, usual quantity per occasion and consumption from HED occasions.*p-value for proportion test

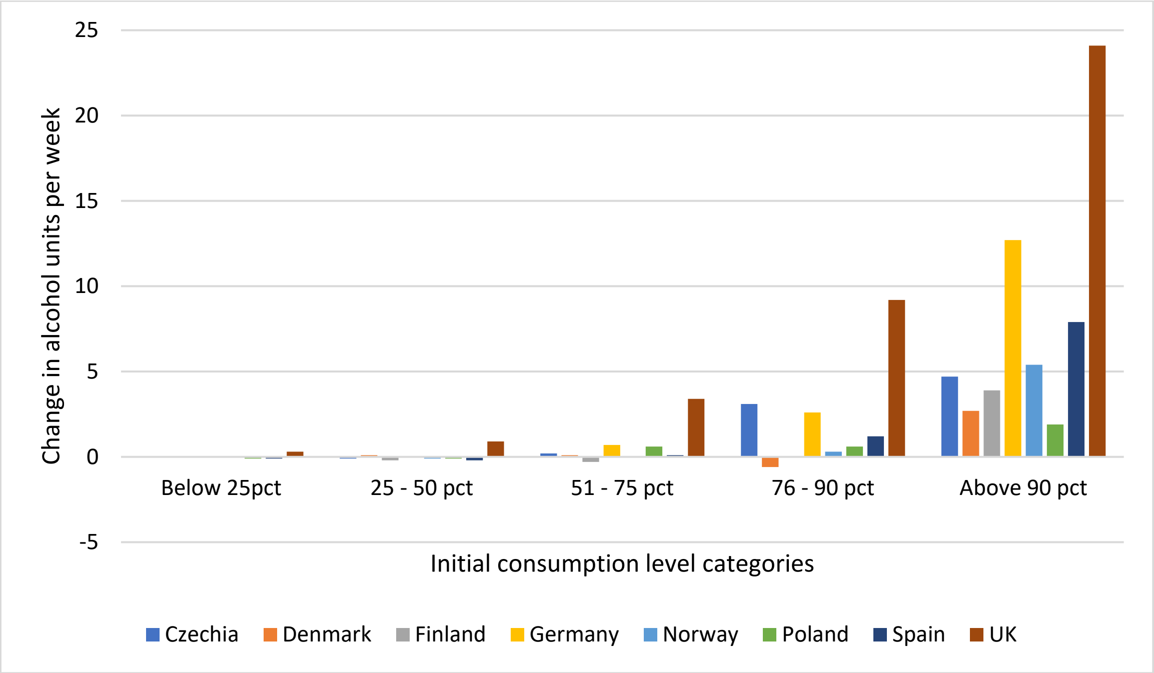

Figure S1A. Change in estimated volume of consumption by initial consumption level and country.

Sensitivity analysis: HED excluded from calculations of initial alcohol consumption and quantified change in alcohol consumption.

Note: Initial consumption categories are based on percentiles of the distribution in each country.

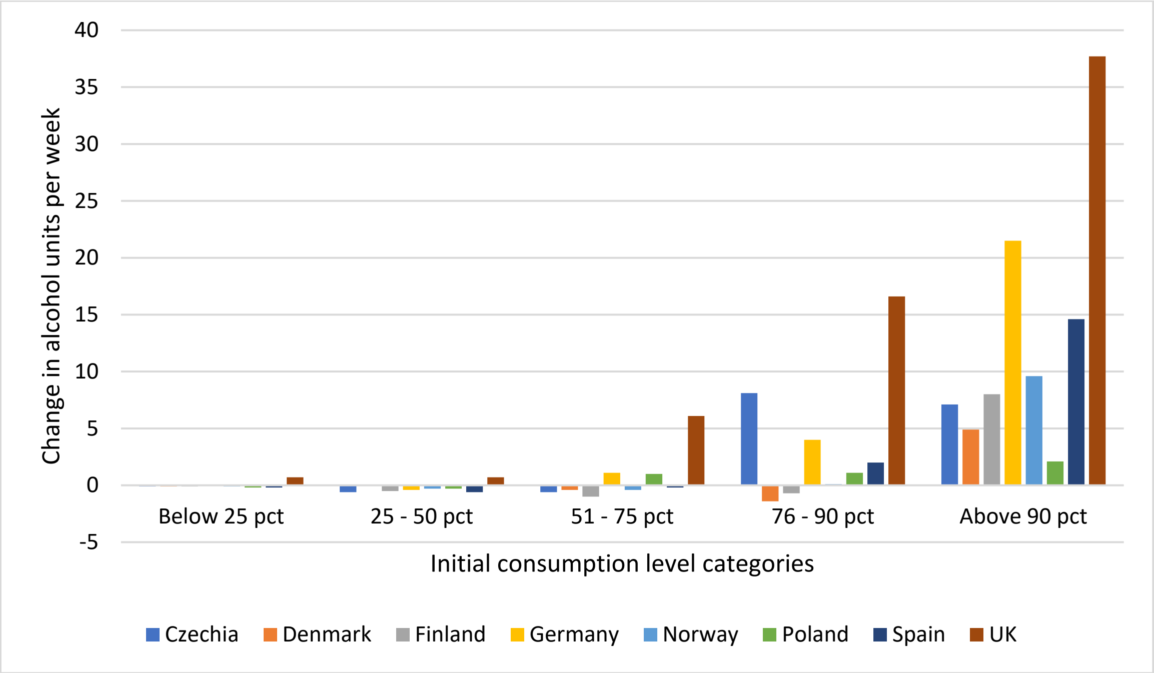

Figure S1B. Change in estimated volume of consumption by initial consumption level and country.

Sensitivity analysis: assuming a larger relative quantified change in drinking frequency, usual quantity per occasion and consumption from HED occasions.

Note: Initial consumption categories are based on percentiles of the distribution in each country.
